# Comparative Genomic Analysis of Rice with Contrasting Photosynthesis and Grain Production under Salt Stress

**DOI:** 10.3390/genes10080562

**Published:** 2019-07-25

**Authors:** Chakkree Lekklar, Duangjai Suriya-arunroj, Monnat Pongpanich, Luca Comai, Boonthida Kositsup, Supachitra Chadchawan, Teerapong Buaboocha

**Affiliations:** 1 Biological Sciences Program, Faculty of Science, Chulalongkorn University, Bangkok 10330, Thailand; 2 Center of Excellence in Environment and Plant Physiology, Department of Botany, Faculty of Science, Chulalongkorn University, Bangkok 10330, Thailand; 3 Molecular Crop Research Unit, Department of Biochemistry, Faculty of Science, Chulalongkorn University, Bangkok 10330, Thailand; 4 Nakohn Ratchasima Rice Research Center, Rice Department, Ministry of Agriculture and Cooperative, Nakohn Ratchasima 30110, Thailand; 5 Department of Mathematics and Computer Science, Faculty of Science, Chulalongkorn University, Bangkok 10330, Thailand; 6 Omics Sciences and Bioinformatics Center, Faculty of Science, Chulalongkorn University, Bangkok 10330, Thailand; 7 Department of Plant Biology and Genome Center, University of California Davis, Davis, CA 95616, USA

**Keywords:** genome, *Oryza sativa* L., photosynthesis, salinity, yield

## Abstract

Unfavourable environmental conditions, including soil salinity, lead to decreased rice (*Oryza sativa* L.) productivity, especially at the reproductive stage. In this study, we examined 30 rice varieties, which revealed significant differences in the photosynthetic performance responses under salt stress conditions during the reproductive stage, which ultimately affected yield components after recovery. In rice with a correlation between net photosynthetic rate (*P*_N_) and intercellular CO_2_ concentration (*C*_i_) under salt stress, *P*_N_ was found to be negatively correlated with filled grain number after recovery. Applying stringent criteria, we identified 130,317 SNPs and 15,396 InDels between two “high-yield rice” varieties and two “low-yield rice” varieties with contrasting photosynthesis and grain yield characteristics. A total of 2089 genes containing high- and moderate-impact SNPs or InDels were evaluated by gene ontology (GO) enrichment analysis, resulting in over-represented terms in the apoptotic process and kinase activity. Among these genes, 262 were highly expressed in reproductive tissues, and most were annotated as receptor-like protein kinases. These findings highlight the importance of variations in signaling components in the genome and these loci can serve as potential genes in rice breeding to produce a variety with salt avoidance that leads to increased yield in saline soil.

## 1. Introduction

Abiotic stresses, including high salinity, are major constraints to crop productivity [1]. Salinity is a term used to describe the presence of different salts, including sodium chloride (NaCl), in soil and water, which has been the subject of intense research [2]. The low water availability under salt stress due to the accumulation of salts such as Na^+^ and Cl^−^ leads to a low water potential gradient between the external environment and the root under early-occurring unfavourable conditions (osmotic phase). Following the first phase, ion toxification caused by the uptake of Na^+^ and Cl^−^ in large amounts by roots negatively affects plant growth by interfering with metabolic processes and decreasing photosynthetic efficiency (ionic stress). The reduction in photosynthesis under salt stress is attributed to either stomatal closure, which leads to a reduction in intercellular CO_2_ partial pressure, or non-stomatal factors including the reduction in chlorophyll synthesis, photosynthetic electron transport reaction, and ribulose-1,5-bisphosphate carboxylase/oxygenase (Rubisco) activity for carbon fixation [3,4,5,6]. Salt-driven photosynthesis reduction via stomatal closure has been studied in many plant species, including rice [7,8], since the closure of stomata under salinity is believed to be an important immediate response strategy for controlling water loss in plants [9]. Photosynthetic parameters, such as the photosynthetic rate, stomatal conductance, stomatal size, the transpiration rate, CO_2_ concentration, and water use efficiency, may directly affect the biomass and grain yields of crops under salt stress. Therefore, the relationships between photosynthesis-related parameters and crop yield are challenging points that need to be addressed under both favourable and unfavourable conditions [10,11,12].

Rice (*Oryza sativa* L.) is the main staple food crop worldwide. As the global population increases, the production of rice needs to increase. Furthermore, dramatic global environmental change poses a great threat to rice productivity [13]. Rice is categorized as a salt-susceptible species, and high salinity is one of the main constraints on its production [13]. Many strategies have been used to generate salt-tolerant rice varieties, such as marker-assisted selection or genetic engineering, by introducing salt-tolerance genes [14].

The published rice reference genome [15,16] and the advancement of high-throughput sequencing technologies known as next-generation sequencing (NGS) provide an opportunity for exploring the genetic diversity among various rice accessions and its utilization in genetic improvement [17,18]. Discovery of a very large number of sequence polymorphisms including single nucleotide polymorphisms (SNPs) and insertions/deletions (InDels) by NGS is one of the most important applications of this technology [19,20,21]. Polymorphism discovery related to functional changes in genes is important for investigating genomic loci responsible for phenotypic and physiological traits [17,18,22]. For identification of polymorphisms related to abiotic stress tolerance, comparative whole-genome analysis was performed on rice with contrasting phosphorus (P) deficiency phenotypes. Approximately 5.1 million polymorphisms were identified in P-sensitive and P-tolerant cultivars, which revealed potential variations in phosphate starvation-responsive genes and genes involved in root architecture [23]. In particular, genome-wide screening for variants in rice with contrasting salt stress tolerance (Pokkali and IR64) was conducted by Jain et al. [24]. Approximately 25% of the polymorphisms identified in this study were detected in genic regions and were nonsynonymous SNPs in 5968 genes, which mostly encode pentatricopeptide repeats, leucine-rich repeats, and protein kinase domains. Furthermore, the whole genome of a salt-tolerant rice cultivar, Godawee, was re-sequenced by the Illumina platform, in which ~2.2 million SNPs and ~480,000 InDels and nonsynonymous SNPs (192,249 SNPs) were identified in 31,287 genes. Twenty-eight salt tolerance-related genes were evaluated, and their coding regions contained 78 nonsynonymous SNPs and 76 synonymous SNPs. A sodium transporter gene, *OsHKT2;1* (Os06g0701700), showed the maximum number of nonsynonymous SNPs (32 SNPs). In particular, the upstream regions of the salt tolerance genes *OsAPx8* (Os02g0553200), *OsMSR2* (Os01g0955100), *OsTIR1* (Os05g0150500), *OsHKT2;3* (Os01g0532600), *OsHKT1;4* (Os04g0607600), and *OsSOS1* (Os12g0641100) showed a high abundance of WRKY cis-acting regulatory elements that bind to WRKY transcription factors (TFs), a superfamily of plant TFs responsible for the regulation of genes responsive to many plant growth and developmental cues, as well as salt stress [25].

The effects of salt stress on the photosynthetic rate of rice have been reported in many studies [26,27,28]. The major cause of grain yield reduction under this stress is the limitation of carbohydrate production driven by photosynthesis and the transport of this biomolecule to spikelets. However, research focusing on the relationship between salt effects and photosynthesis at the reproductive stage, which is severely affected by salt stress in terms of yield productivity, has been limited [8,29,30]. To date, NGS technology has been widely used to identify polymorphisms in genes or loci associated with the complex trait of salt-stress responsiveness in rice. In this study, whole-genome sequences of rice varieties with contrasting yield production under salt stress at the reproductive stage, which were selected based on the correlation between photosynthesis parameters and grain yield when exposed to saline soil, were analysed. This analysis revealed numerous DNA polymorphisms controlling the trait, which can be used in combination with high-throughput genotyping in molecular breeding to improve rice salinity tolerance in future studies.

## 2. Materials and Methods

### 2.1. Plant Material and Experimental Design

Seeds of 30 rice (*Oryza sativa* L.) varieties were provided by the Pathum Thani Rice Research Center, Pathum Thani, Thailand. Twenty-nine varieties were local varieties originating in Thailand, and one local landrace (Pokkali) was from India, which was used as a salt-tolerant variety. The experiment was conducted, as described by Lekklar et al. [30] at the Nakhon Ratchasima Rice Research Center, Nakhon Ratchasima Province, Thailand. Rice seeds were soaked for 24 h and germinated on plastic mesh floating on water for 3 days. The seedlings were grown in containers with 1/2-strength modified WP no. 2 nutrient solution for 4 days before being cultured in full-strength nutrient solution for 14 days [31]. The 21-day-old seedlings were then transplanted into pots (four seedlings per pot) filled with 5 kg of soil. The pot soil was maintained under flooded conditions (2–3 cm of water above the soil surface) during the rice growth period. After 1 and 2 months, the plants were applied with 15-15-15 chemical fertilizer (Rabbit, Chia Tai Co., Ltd., Thailand). At the flowering stage, rice was treated by adding 150 mM NaCl solution to the soil (900 mL per pot) after water was drained out. The soil was submerged in NaCl solution, approximately 2 cm above the soil surface to reach the desired final soil electrical conductivity (EC) of ~8 dS/m for 9 days, whereas tap water was added for the normal condition. For recovery to normal conditions, tap water was added to the pot, and the pot was drained for a week until the EC in the soil equalled ~2 dS m^−1^. The crops were then harvested at maturity. Four replicate pots of each variety were planted in a randomized complete block design.

### 2.2. Photosynthetic Parameter and Grain Yield Measurement

Leaf photosynthesis characteristics were measured on the penultimate leaf (the 2nd leaf from the flag leaf) of the main tiller from 8.30 to 11.30 a.m. 0, 3, 6, and 9 days after treatment using a portable photosynthesis system (LI-COR 6400-XT, LI-COR, Nebraska, USA) with a red-blue light source and a 2 × 3 cm^2^ cuvette as described by Lekklar et al. [30]. The cuvette conditions were as follows: PAR, 1200 μmol m^−2^ s^−1^; flow rate, 500 μmol s^−1^; sample CO_2_, 380 μmol mol^−1^; and leaf temperature, 30 ± 5 °C, which equalled the ambient temperature. Photosynthetic parameters consisted of the net photosynthesis rate (*P*_N_), stomatal conductance (*g*_s_), the transpiration rate (*E*), and intercellular CO_2_ concentration (*C*_i_). After rice plants recovered from salt stress conditions, the filled grain number (FG) was recorded from 4 plants of each of the 30 varieties.

### 2.3. Statistical Analysis

The experimental design was a randomized complete block design. The results were expressed as the mean ± standard error (SE). Analysis of variance (ANOVA) was used to evaluate the data for each parameter. Differences among the means were compared by Duncan’s multiple range test (DMRT). Differences were considered statistically significant when *p*-value < 0.05. The Pearson correlation coefficients (*r*) among photosynthetic parameters and yield components were determined using JMP software ver. 9 (SAS, Cary, NC, USA).

### 2.4. DNA Sequencing, Mapping and Variant Detection

The total DNA of each rice variety was extracted from young leaf tissue with a Genomic DNA Mini Kit (Plant) (Geneaid Biotech Ltd., Taiwan). Whole-genome library preparation was performed, as described by Lekklar et al. [30]. The IRGSP-1.0 [32] rice reference genome was downloaded from the Rice Genome Annotation Project web page (http://rice.plantbiology.msu.edu). We used the pipeline created by Missirian et al. [33] to demultiplex sequenced reads from different libraries. Raw reads were aligned to the reference genome using the Burrows-Wheeler Aligner (BWA version 0.5.7-1) [34]. Genome Analysis Toolkit (GATK; version 3.3–0) was employed with default parameters to identify variants (GATK; version 3.3–0) [35].

### 2.5. Variant Annotation

After variant calling, SNPs and InDels were further filtered to retain good-quality variants with a read depth ≥ 10 and genotype quality score ≥ 40. SnpEff was used to annotate SNPs and InDels [36], which annotates variants by sequence ontology, nucleotide substitution, and region on the rice genome.

### 2.6. Ontology Enrichment Analysis and Expression Profile of Candidate Genes

We used a web-based platform, Comprehensive Annotation of Rice Multi-Omics (CARMO) [37], to identify enriched gene ontologies, which were based on a 5% false discovery rate (FDR). The GO enrichment results were visualized by ‘ggplot2’ in R [38]. The same platform was also used to evaluate the tissue-specific expression of candidate genes.

## 3. Results

### 3.1. Variation in and Correlations between Photosynthetic Performance Parameters of 30 Rice Varieties

**Figure 1:**
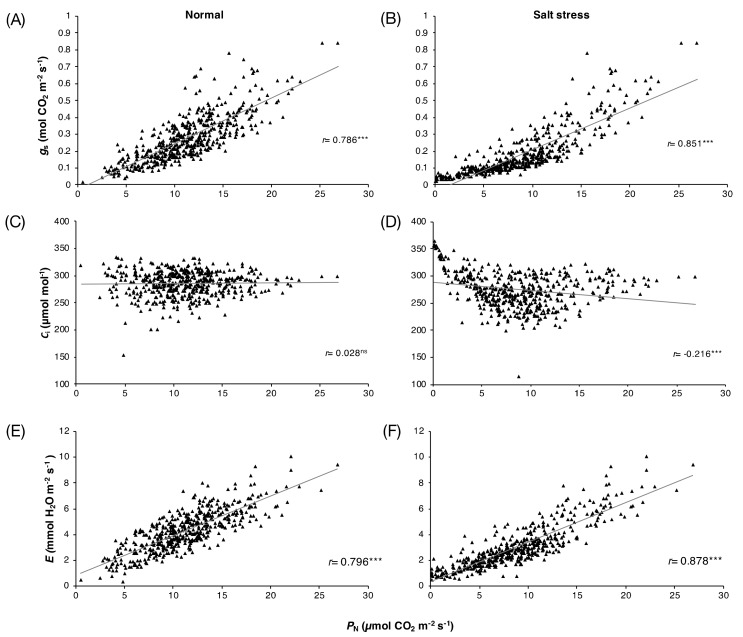
Correlations among photosynthetic parameters of 30 rice varieties grown under normal and salt stress conditions for 0, 3, 6, and 9 days: *P*_N_ and *g*_s_ (**A**,**B**), *P*_N_ and *C_i_* (**C**,**D**) and *P*_N_ and *E* (**E**,**F**).

The photosynthetic parameters (*P*_N_, *g*_s_, *C*_i_, and *E*) of 30 rice varieties (Table S1) at the flowering stage grown under normal and salt stress conditions for 3, 6, and 9 days are shown in Table S2. The results revealed significant differences in the photosynthetic performance responses of these rice genotypes under salt stress conditions. On day 9 of the treatment, under normal conditions, the *P*_N_ in all varieties was in the range of 3.57–13.38 μmol CO_2_ m^−2^ s^−1^, while under salt stress conditions, the *P*_N_ of these varieties was in the range of 0.4–712.57 μmol CO_2_ m^−2^ s^−1^. Salt stress substantially reduced the *P*_N_ in all rice varieties by day 9 of treatment, except for ‘Sam Ahang’ rice, which had a higher *P*_N_ under salt stress than under normal conditions. The results of the correlation analysis of the four photosynthetic parameters under normal and stress conditions on days 0, 3, 6, and 9 of the 30 rice varieties are presented in Figure 1. Under normal conditions, significant positive correlations were found between *P*_N_ and *g*_s_, *r* = 0.786 (*p*-value < 0.001) (Figure 1A), and between *P*_N_ and *E*, *r* = 0.796 (*p*-value < 0.001) (Figure 1E). Similarly, under salt stress conditions, significant positive correlations between *P*_N_ and *g*_s_, *r* = 0.851 (*p*-value < 0.001) (Figure 1B), and *P*_N_ and *E*, *r* = 0.878 (*p*-value < 0.001) (Figure 1F), which were stronger than those under normal conditions, were found. Interestingly, no correlation between *P*_N_ and *C*_i_ was observed under normal conditions (Figure 1C). However, a significant negative correlation between these parameters was found under salt stress conditions, *r* = −0.216 (*p*-value < 0.001) (Figure 1D).

### 3.2. Clustering Rice Varieties Using Differences in the Correlation between P_N_ and C_i_

The correlation of photosynthetic parameters varied among the varieties examined. The correlations between *P*_N_ and *g*_s_, *C*_i_ or *E* in individual varieties under normal and salt stress conditions are presented in Table 1. Under normal condition, positive correlations between *P*_N_ and *g*_s_ were found in all varieties except ‘Sam Ahang’; between *P*_N_ and *E*, positive correlations were found in all varieties except ‘Hahng Nahk’, ‘Sam Ahang’, ‘Mahk Yom’, and ‘Mahk Bid’. However, no correlation between *P*_N_ and *C*_i_ was found in most varieties under normal conditions. Under salt stress conditions, significant correlations were found in all varieties between *P*_N_ and *g*_s_, *r* = 0.70 (*p*-value < 0.001) to *r* = 0.96 (*p*-value < 0.001), as well as between *P*_N_ and *E* (*r* = 0.57, *p*-value < 0.05 to -*r* = 0.97, *p*-value < 0.001). Interestingly, a correlation between *P*_N_ and *C*_i_ under salt stress conditions was found in 10 rice varieties, namely, ‘Ma Yom’, ‘Tah Bahn’, ‘Khitom Khao’, ‘Leuang Dong’, ‘Mae Mai’, ‘Jao Khao’, ‘Di Si’, ‘Med Makham’, ‘Nahng Nuan’, and ‘Sew Mae Jan’. Therefore, based on the correlation between *P*_N_ and *C*_i_ under salt stress conditions, 10 varieties were categorized as ***group I*** and the other varieties (20 varieties) that had no correlation between *P*_N_ and *C*_i_ were categorized as ***group II*** to further examine the correlation between *P*_N_ and yield components.

### 3.3. Correlation between P_N_ and Grain Yield of the Rice Groups

**Figure 2:**
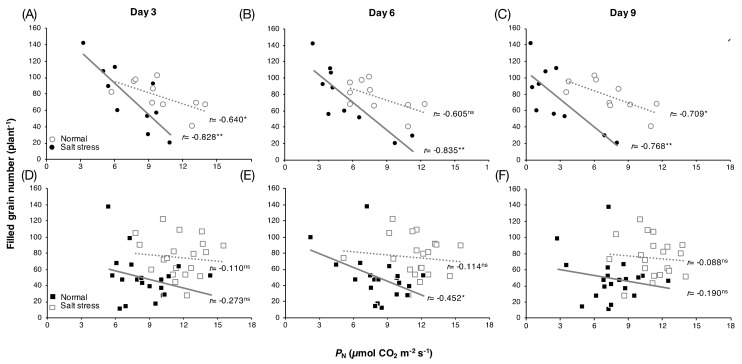
Correlation between leaf *P*_N_ and grain yield per plant of *group I* rice (**A**–**C**) and group II rice (**D**–**F**) grown under normal and salt stress conditions for 3, 6 and 9 days, respectively. *significant at *p*-value < 0.05, **significant at *p*-value < 0.01, and ns = not significant.

Among ***group I*** rice, significant negative correlations between *P*_N_ and grain yield under salt stress conditions were found at all time points examined: Day 3 (Figure 2A), day 6 (Figure 2B), and day 9 (Figure 2C), with *r* = −0.828 (*p*-value < 0.01), *r* = −0.835 (*p*-value < 0.01), and *r* = −0.768 (*p*-value < 0.01), respectively. Under normal conditions, weaker negative correlations on day 3 (Figure 2A) and day 9 (Figure 2C), with *r* = −0.640 (*p*-value < 0.05) and *r* = −0.709 (*p*-value < 0.05), respectively, and no correlation on day 6 (Figure 2B) were observed. For ***group II*** rice, under salt stress conditions, no correlation between these parameters on day 3 (Figure 2D) and day 9 (Figure 2F) and a relatively weak negative correlation on day 6, *r* = −0.452 (*p*-value < 0.05) (Figure 2E), were found. Under normal conditions, no correlation was observed for ***group II*** rice at any time point examined (Figure 2D–F).

### 3.4. Whole-Genome Resequencing Analysis and Variant Discovery

**Figure 3:**
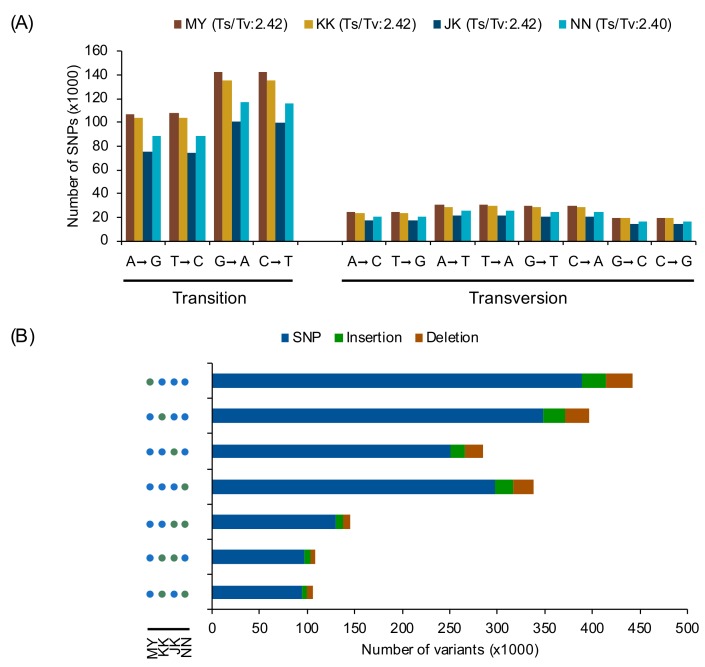
(**A**) Nucleotide substitution frequency of each rice genome as determined by SnpEff [36]. (**B**) Number of variants in each LYR and HYR variety. Green and blue circles indicate that corresponding varieties share the variants. MY: Ma Yom, KK: Khitom Khao, JK: Jao Khao, NN: Nahng Nuan.

Four rice varieties from ***group I***, which exhibited correlations between *P*_N_ and filled grain (Figure 2), were selected for whole-genome sequencing. We selected 2 salt-affected “low-yield rice (LYR)” varieties (‘Ma Yom’, MY, and ‘Khitom Khao’, KK) and 2 “high-yield rice (HYR)” varieties (‘Jao Khao’, JK, and ‘Nahng Nuan’, NN) [30]. The high-quality, 150-bp-long paired-end reads from each variety were aligned to the reference genome ‘Nipponbare’, separately. More than 30 million reads were obtained from each variety, and the resulting mapping rate ranged from 89.52%–95.07%. We found a total of 707,759, 678,820, 497,512, and 583,761 SNPs in ‘MY’, ‘KK’, ‘JK’, and ‘NN’, respectively. Furthermore, 89,400, 85,700, 61,385, and 73,471 InDels were identified in the 4 rice varieties, respectively (Table 2). For nucleotide substitution comparison, all SNPs were subdivided into transitions (Ts) and transversions (Tv). For Ts, G→A and C→T were found more frequently than A→G and T→A in all varieties (Figure 3A). For Tv, T→A was the most frequent, followed by A→T, G→T, and C→A, which were found at similar frequencies in all varieties, while G→C was the least frequent in all varieties. The range of the Ts/Tv ratio for all rice varieties was 2.40–2.42. These results suggest that the substitution patterns in these rice varieties were similar. The regions in which all polymorphisms were located in each rice variety are summarized in Table S3. The majority of SNPs in all rice varieties were located in upstream (~35%), downstream (~34%), or intergenic (~23%) regions. Similarly, we found a large number of InDels located in the upstream and downstream regions in all rice varieties (Table S3). Approximately 8% of SNPs and InDels were found in genes, approximately half of which were found in introns. In total, approximately 3% of SNPs and 2% of InDels were located in expressed regions.

### 3.5. Structural and Functional Annotation of Variants between LYR and HYR

To compare the variant-sharing profiles of the four rice varieties, variants were analysed using the combined and selected variants function in GATK [35]. We grouped all positions of SNPs shared among the four varieties as variety-specific (defined as a variant not shared by other varieties) or shared by any two varieties. For variety-specific groups, the numbers of positions in the 2 LYR varieties (MY and KK) were larger than those in the 2 HYR varieties (JK and NN) (Figure 3B). For SNPs shared by any two varieties, the number of positions shared by LYR or by HYR was 145,713, which was larger than that shared by the other groups of two varieties (Figure 3B and Figure S1).

Moreover, we predicted the effects of variants on protein function, which were clustered into four types (high, moderate, low, and modifier) based on the predicted severity of each effect (Table S4). Most variants belonged to the modifier category, such as the 3′-UTR, 5′-UTR, synonymous SNP and intron variants, which were inferred to have only a weak impact. Nonetheless, numerous variants with high or moderate effects were found among the four varieties. Among those shared between two varieties, the number of SNPs shared by LYR and shared by HYR (MY/KK-JK/NN) was largest in all categories. In the high- or moderate-effect group, a total of 5842 positions were found in the MY/KK-JK/NN group, which was much larger than that found in the MY/JK-KK/NN and MY/NN-KK/JK groups (4133 and 3841, respectively) (Table S4).

### 3.6. Distribution of LYR- and HYR-Shared Variants Detected on Rice Chromosomes

**Figure 4:**
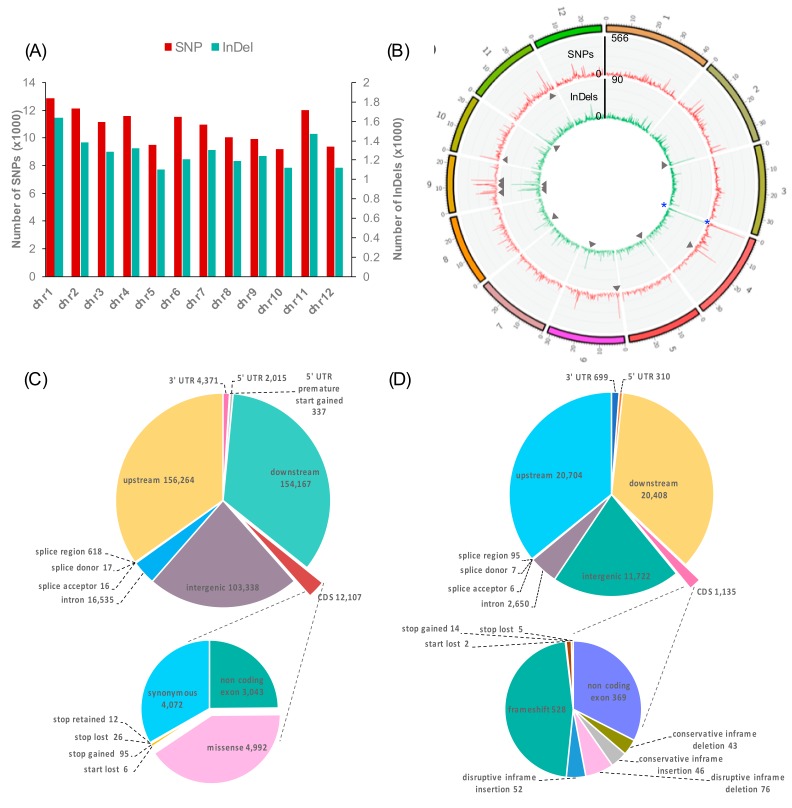
Distribution (**A**) and density (**B**) of variants across rice chromosomes identified in MY/KK–JK/NN. Variant densities were computed within a 100-kb window. The outside and inside of the Circos diagram represent SNP density (red line) and InDel density (green), respectively. The grey arrowheads indicate variant hotspots. MY: Ma Yom, KK: Khitom Khao, JK: Jao Khao, NN: Nahng Nuan. Structural annotation of variants identified in MY/KK-JK/NN. Pie charts represent variant annotations and numbers of MY/KK-JK/NN SNPs (**C**) and InDels (**D**).

The numbers of all variants including the HYR- and LYR-shared SNPs and InDels (MY/KK-JK/NN) were plotted across all rice chromosomes (Figure 4A). The largest numbers of SNPs (12,882) and InDels (1633) were detected on chromosome 1, which was found to be directly proportional to chromosome length. However, the highest densities of SNPs (42.8 SNPs/100 kb) and InDels (5.3 InDels/100 kb) were found on chromosome 9 (Table S5). Figure 4B represents the frequency of the HYR- and LYR-shared variants calculated within a 100-kb window size using VCFtools [39] and visualized by ClicO FS [40]. Surprisingly, the highest densities of HYR- and LYR-shared SNPs (566) and InDels (90) were identified on chromosome 4. For regions of hotspots, which contained ≥ 300 SNPs/100 kb or ≥ 40 InDels/100 kb for the HYR- and LYR-shared variants, 8 SNP hotspots were found on chromosomes 4, 6, 9, 10, and 11, and 8 InDel hotspots were found on chromosomes 2, 5, 6, 8, 9, and 11 (Figure 4B).

### 3.7. Characteristics of LYR- and HYR-Shared Variants

The majority of the LYR- and HYR-shared SNPs (MY/KK-JK/NN) were identified in upstream (156,264), downstream (154,167), and intergenic regions (103,338). Within genic regions, we identified 16,535 SNPs in introns, 6723 SNPs in UTRs, and 651 SNPs in splice sites. A total of 12,107 SNPs were located in coding sequences (CDSs), among which 4072 were synonymous SNPs and 4992 were missense (non-synonymous) SNPs, which are variants causing changes in amino acids in proteins. Notably, 95 stop-gained SNPs were identified, resulting in premature stop codons and leading to disrupted transcription of genes (Figure 4C). 

In total, 20,704, 20,408, and 11,722 InDels were identified in upstream, downstream, and intergenic regions, respectively. Within genic regions, we identified 2650 InDels in introns, 1,009 InDels in UTRs, and 108 InDels in splice sites. A total of 1135 InDels were located in CDSs, among which 528 caused a frameshift; 52 caused disruptive in-frame insertion; and 76 caused disruptive in-frame deletion. These annotated variants caused disruption of the translational reading frame (Figure 4D).

### 3.8. GO Enrichment Analysis of Genes Containing High- and Moderate-Impact Variants

**Figure 5:**
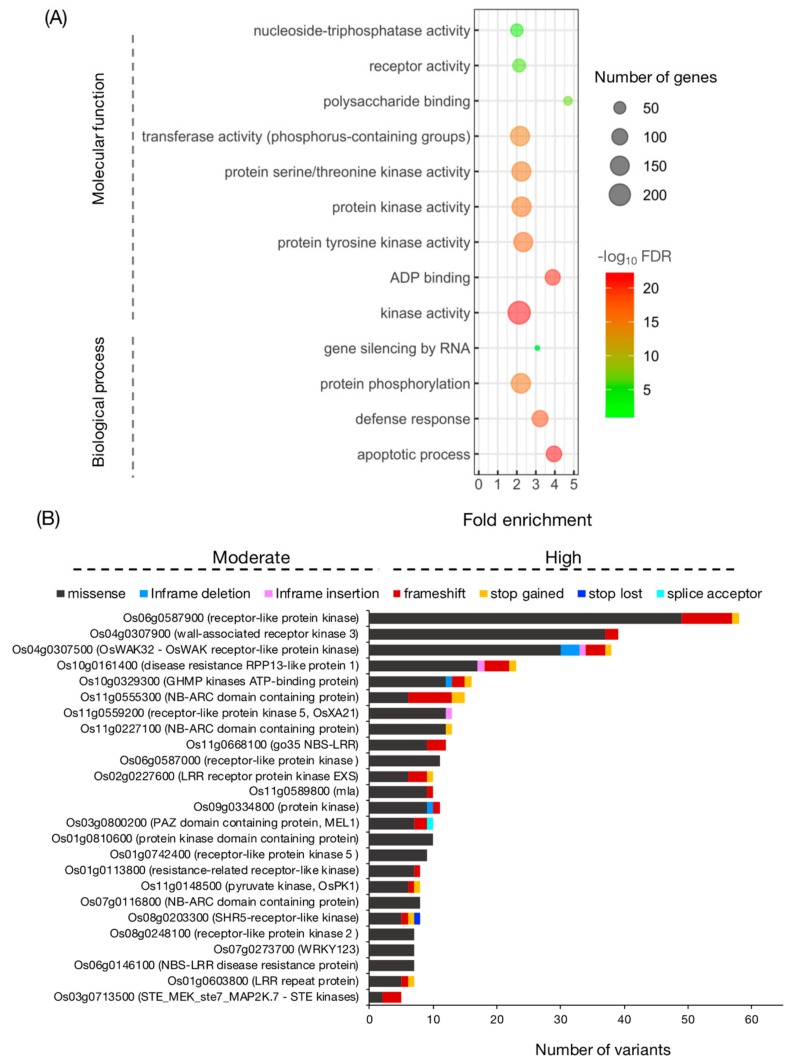
Gene ontology enrichment analysis of genes containing high- and moderate-impact variants identified in MY/KK-JK/NN. Darker colours represent higher significance, and larger circles indicate more enriched genes in the group (**A**). Bar diagram showing genes containing the most high- and moderate-impact SNPs/InDels identified in MY/KK-JK/NN (**B**). MY: Ma Yom, KK: Khitom Khao, JK: Jao Khao, NN: Nahng Nuan.

To explore potential variations in protein function, we focused on high- and moderate-impact variants of the HYR- and LYR-shared SNPs and InDels. A list of 2089 genes containing high- and moderate-impact variants is presented in Table S6. These genes were submitted to the ‘CARMO’ GO enrichment facility [37]. The significance level is based on Fisher’s exact test and multi-test adjustment using a 5% false positive detection (FDR) threshold (Table S7). The results revealed that in terms of biological process, there were 4 enriched GOs, namely, the apoptotic process (GO:0006915), defence response (GO:0006952), protein phosphorylation (GO:0006468), and gene silencing by RNA (GO:0031047) (Figure 5A). In terms of molecular function, there were 9 enriched GOs, namely, 4 GOs involving kinase activity (GO:0016301, GO:0004713, GO:0004672 and GO:0004674) and on each involving ADP binding (GO:0043531), transferase activity (GO:0016772), polysaccharide binding (GO:0030247), receptor activity (GO:0004872), and nucleoside-triphosphatase activity (GO:0017111). Notably, most genes containing high- and moderate-impact variants were over-represented by GO terms in kinase activity (Figure 5A).

### 3.9. Potential Genes Containing a Large Number of High- and Moderate-Impact Polymorphisms

From the GO enrichment analysis, we found that 378 genes containing high- and moderate-impact variants were enriched (Table S7). These genes were narrowed down to identify potential genes involved in salt tolerance during the reproductive stage by evaluating their expression profiles in reproductive tissues based on CARMO, a web-based platform [37]. The genes expressed in reproductive tissues, which included the post-emergence inflorescence, pre-emergence inflorescence, anther, and pistil and panicle, were evaluated as potential genes that may be involved in yield productivity during salt stress. We identified 262 genes highly expressed in these reproductive tissues (Figure S2 and Table S8). A list of the top 10 highest-expressing genes in any one of the reproductive tissues examined is shown in Table 3. A large number of variations were identified in OS01G0810600 (protein kinase domain-containing protein), OS03G0800200 (PAZ domain-containing protein, *OsMEL1*), OS05G0596600 (RecF/RecN/SMC N terminal domain-containing protein), OS11G0148500 (pyruvate kinase, *OsPK1*), and OS11G0227100 (NB-ARC domain-containing protein).

Furthermore, Figure 5A shows the top 25 genes with the most high- and moderate-impact variants and that were highly expressed in reproductive tissues (Figure S2). Most of the identified genes belong to the receptor-like kinase (RLK) family. The largest number of variations was found in Os06g0587900 (receptor-like protein kinase), which harboured 49 missense SNPs, 8 frameshift InDels, and 1 stop-gained SNP. A total of 37 missense SNPs and 2 frameshift InDels were found in Os04g0307900 (wall-associated receptor kinase 3). In addition, 30 missense SNPs, 3 in-frame deletion InDels, 3 frameshift InDels, 1 in-frame insertion InDel, and 1 stop-gained InDel were identified in Os04g0307500 (OsWAK32).

Previous reports described the isolation and characterization of 3 genes found here, namely, *OsXA21* (Os11g0559200), *OsMEL1* (Os03g0800200), and *OsPK1* (Os11g0148500) (Figure 5B). A total of 14 variants (12 missense SNPs, 1 in-frame deletion, and 1 in-frame insertion) were identified in *OsXA21*, which confers resistance to *Xanthomonas oryzae pv. oryzae* [41]. *OsMEL1*, a PAZ domain-containing protein, has been reported to be a key gene involved in meiosis in rice germ cells and involved in a gene-regulatory system via small RNA-mediated gene silencing in rice sexual reproduction [42]. This gene contained 7 missense SNPs, 1 splice acceptor SNP, and 2 frameshifts. Finally, a pyruvate kinase named *OsPK1* showed 6 missense SNPs, 1 frameshift InDel, and 1 stop-gained SNP. Mutation of these genes causes dwarfism and panicle enclosure in rice [43] (Figure S3).

## 4. Discussion

### 4.1. Salt-Affected Photosynthetic Characteristics of Flowering Rice Exposed to Saline Soil

Upon salt stress, many major physiological processes are negatively affected, including photosynthesis, the key biochemical process through which CO_2_ and water are converted into O_2_ and through which the energy-rich sugar compounds that fuel plant growth are synthesized [3,4]. In rice, the severity of injury from salt stress depends on the growth stage, as the most sensitive periods are the seedling and reproductive stages [44,45]. In this study, we focused on the reproductive stage, in which photosynthetic capacity directly affects grain yield. The positive correlation between *P*_N_ and *g*_s_ found in all rice varieties under salt stress (Table 1) in rice exposed to salinity may be because salt is effective in mediating stomatal closure via the accumulation of abscisic acid (ABA), a plant phytohormone that accumulates in stressed roots in saline soil and is transported to the aerial part via xylem [46,47]. The observed relationship between *P*_N_ and *g*_s_ has been reported in rice cultivars in diverse growing environments, including upland and lowland rice during flowering time in the penultimate leaf of the main tiller, similar to this study [48]. Consistently, because *g*_s_ directly controls the transpiration from leaf to the ambient air in plants [49,50], a positive correlation was found between *P*_N_ and *E* in all varieties (Figure 1 and Table 1). However, only 10 varieties showed a correlation between *P*_N_ and *C*_i_ under salt stress conditions. Most showed a negative correlation, except ‘Ma Yom’ and ‘Sew Mae Jan’, which showed a positive correlation between these parameters. *C*_i_, the CO_2_ concentration in the intercellular airspace in a plant leaf, can reflect (1) the amount of ambient CO_2_ that diffuses to the stomatal pore on the leaf surface and (2) the activity of Rubisco enzyme for CO_2_ fixation under salt stress [4,51,52]. Furthermore, in C_3_ plants, including rice, mesophyll conductance to allow CO_2_ diffusion (*g*_m_) has been investigated as a key limiting factor of photosynthesis [53,54]. *g*_m_ describes the movement of CO_2_ from intercellular space to mesophyll cells via the cell walls, plasma membrane, cytoplasm, and chloroplast envelope to the part of the chloroplast stroma where photosynthesis is taking place, which accounts for the CO_2_ in the chloroplast stroma [55,56]. This parameter was reported to be related to mesophyll structural traits in rice such as cell wall thickness (*T*_w_) and mesophyll cell surface area exposed to intercellular air space per leaf area (*S*_m_) [57,58]. In rice cultivars exposed to drought stress, Ouyang et al. [48] investigated the relationship between leaf anatomy and *g*_m_. Particularly, they found a positive correlation between *g*_m_ and the ratio of the exposed chloroplast surface area (*S*_c_) to *S*_m_, suggesting the importance of these parameters in the relationships among photosynthetic parameters in response to abiotic stress in rice [59,60]. As many parameters are involved in photosynthetic performance in rice, in this study, two groups of rice varieties under salt stress, one with a relationship between *P*_N_ and *C*_i_ and the other without, were observed, suggesting genotype-dependent differences in the observed phenotypic data between the two rice groups (Table 1 and Figure 2).

### 4.2. Prediction of Grain Yield by Photosynthetic Performance and Salt-Stressed Flowering Rice

Enhancement of plant photosynthesis to improve grain yield is an extensively accepted strategy for meeting the global food demand [10,61,62]. For rice, much evidence obtained by various procedures, such as canopy architecture modification, C_4_ pathway introduction and photorespiration manipulation, has revealed a strong relationship between enhanced photosynthesis and yield [63,64,65,66]. However, little attention has been given to the evaluation of high-photosynthetic-performance rice and its correlation between photosynthetic parameters and yield components under salt stress. In fact, salt-induced stress during the reproductive stage of rice can lead to a decline in yield parameters such as pollen viability, grain number, and grain weight (see the review by Hussain et al. [29]). While the response of rice photosynthesis to salt stress has been reported, only a few studies have evaluated photosynthetic performance together with grain yield. In these studies, the decrease in rice yield under salt stress, particularly due to inhibition of biomass accumulation, was reported to be associated with a decreasing rate of photosynthesis. However, rice cv. Bahia exhibited different physiological responses under salt stress at the reproductive stage. Under salinity, this cultivar had a similar *P*_N_ to that under normal conditions, but exhibited salt-induced panicle sterility [67]. Therefore, the relationship between the photosynthetic rate and yield parameters of rice under salt stress is still unclear. Contrary to several previous studies, among rice groups based on *C*_i_ under salt stress in this study, a large filled grain number of rice plants was found in rice exhibiting a low *P*_N_, while a small filled grain number was found in rice exhibiting a high *P*_N_ (Figure 2, Figure S4 and Figure S5). One possible physiological response-related explanation of this result is based on the transport of salt ions. Generally, salt ions are taken up from saline soil by roots and transported to the aerial parts of plants through the transpiration stream [68,69]. Therefore, restriction of Na^+^ accumulation in the shoot can presumably be achieved by reducing transpiration through stomatal movement regulation, as observed in the high-yield rice (HYR: Jao Khao, JK, and Nuang Nuan, NN), while the opposite was found in the low-yield rice (LYR: Ma Yom, MY, and Khitom Khao, KK) (Figures S4 and S5).

### 4.3. Validation of Genome-Wide Sequence Variants Revealed Potential Genes

To elucidate genome-wide DNA polymorphisms in the four rice varieties that exhibited contrasting photosynthetic performances and filled grain numbers under salt stress at flowering time, whole-genome sequencing of each rice variety was performed via the Illumina sequencing platform, resulting in an average of 34.6 × 10^6^ high-quality reads (150-bp paired-end reads). Overall, ~93% of the qualified reads were mapped to the reference genome (cv. Nipponbare). The variation in variant numbers across rice varieties appeared to correspond to the number of sequenced reads (Table 2). For nucleotide substitution analysis, the ratio of transitions to transversions (Ts ⁄ Tv) of ~2.4 (Figure 3A) was found. This phenomenon is known as ‘transition bias’, which has previously been reported in rice [70]. The Ts/Tv ratio observed here is higher than that in a previous study in rice [24,71,72]. A higher Ts/Tv ratio is indicative of a low level of genetic divergence. These ratios are expected to decline with increasing genetic distance between the compared genotypes with time; transversions erase the record of frequent transitions [23,73].

With an in-depth analysis of the potential polymorphisms responsible for filled grain number under salt stress of flowering rice, the annotation of polymorphisms observed in MY/KK-JK/NN revealed that only ~3% of SNPs and ~2% of InDels were detected in CDSs, whereas the remaining ~35% of SNPs and ~36% of InDels were detected in upstream regions (Figure 4C,D). The larger number of variations in regulatory regions other than genic regions found in this study corresponds to the results of previous studies in rice [74,75]. We identified 722 high-impact variants resulting in changes such as frameshifts, exon loss and stop loss and 5120 moderate-impact variants, such as missense variants (non-synonymous) (Table S4). Overall, a total of 2089 genes harbouring at least one large-effect SNP and/or InDel were identified (Figure 4C,D and Table S6). The genes encoding receptor-like kinases were most represented in this study (Table S6).

For GO enrichment analysis (Figure 5A), most of the genes enriched in apoptotic process (FDR < 1.86994 × 10^−22^) encode NB-ARC (nucleotide-binding adaptor shared by APAF-1, R proteins) proteins and NBS-LRR (nucleotide-binding site leucine-rich repeat) proteins. While salt tolerance is an abiotic trait, Nejat and Mantri [76] suggested that plant innate immunity evolved to respond to the crosstalk between multiple biotic and abiotic stresses. NBS-LRR or NB-ABC domain-containing proteins may recognize pathogen-associated molecular patterns (PAMPs) during the abiotic stress response. The perception of PAMPs is crucial during pathogen attack [77], but plants may also sense abiotic stress by surface-localized pattern recognition receptors (PRRs) [78,79]. Kinase activity (FDR < 5.31095 × 10^−22^) was the most significant GO term in the molecular function category, and almost all were described as receptor-like kinases (RLKs). It has been proposed that the diversity of the extracellular domains in plant RLKs reflects their importance in rapidly evolving to defend against an ever-changing population of ligands produced by abiotic stresses in plants [80]. Thus, the high variation detected in this study likely reflects variation between the LYR and HYR in signalling pathway components.

Among the genes that were enriched in GO terms, we found that a total of 250 genes were highly expressed in reproductive tissues, narrowing down the genes involved in grain yield in rice (Figure S2, Table S8). Notably, three genes were cloned and characterized, namely, *OsXA21*, *OsMEL1,* and *OsPK1* (Figure S3), and two of these were also in the top 10 most highly expressed genes in any one of the reproductive tissues (Table 3)*. OsXA21*, a member of the leucine-rich repeat receptor-like kinase (LRR-RLK) family, is responsible for resistance to *Xanthomonas oryzae* pv. *oryzae* (*Xoo*), which causes bacterial leaf blight [41,81]. This gene encodes an immune receptor kinase whose functions result in subsequent activation of an intracellular defence response. Additionally, a previous study found that silencing *OsXA21* led to rice susceptibility to *Xoo* infection [82]. Recently, Ye et al. [80] reported that many RLKs are involved in responses to abiotic stresses, including the salt-stress response in plants. Approximately 1,000 RLK genes have been identified in rice, which can be classified into 16 sub-groups. The most abundant RLK in the rice genome is LRR-RLK [83,84]. Gao and Xue [83] reported that many of the rice RLK genes are regulated by salt stress. One example is a rice putative RLK gene, *OsSIK1*, which is mainly induced by salt, drought, and H_2_O_2_ treatments. Rice over-expressing *OsSIK1* showed higher tolerance to salt via increased antioxidant capacity than WT (‘Nipponbare’) rice, an RNAi line and a mutant line. A total of 10 large-impact polymorphisms were identified in *OsMEL1* (MEIOSIS ARRESTED AT LEPTOTENE1), which controls the cell division of premeiotic germ cells. This gene was previously demonstrated to be essential for sporogenesis in rice anthers and has been shown to associate preferentially with 21-nucleotide phased small-interfering RNAs (siRNAs). Loss of MEL1 function resulted in complete seed sterility [42,85]. The role of siRNAs in abiotic stress, including salt stress, in plants has been reported to involve epigenetic regulators of gene expression and post-transcriptional gene silencing. OsMEL1 is a PAZ domain-containing protein that interacts with the argonaute protein to form RISCs (RNA-induced silencing complexes), causing the silencing of the target gene by transcript cleavage or translational suppression [86,87,88]. Furthermore, *OsPK1,* a cytosolic pyruvate kinase, was identified to contain 6 missenses, one frameshift and one stop-gained variant. It is a key regulatory enzyme of the glycolytic pathway that catalyses the final step of glycolysis, converting ADP and phosphoenolpyruvate (PEP) to ATP. This biochemical reaction is irreversible transphosphorylation [89]. Moreover, its substrate, PEP, and product, pyruvate, are both involved in a variety of cellular metabolic fluxes controlling plant growth and development [90,91]. Mutation of *OsPK1* causes dwarfism and panicle enclosure in rice. Furthermore, *ospk1* mutant rice displayed higher levels of H_2_O_2_ in the flag leaf than the WT, suggesting that this gene is involved in the oxidative stress response [43]. However, the role of *OsPK1* in the salt stress response has not been reported. Recently, the interaction between pyruvate kinase (PK) and ENO2 (enolase2) was reported in Arabidopsis. The *eno2* mutant showed complete susceptibility to salt stress [92]. Moreover, *ENO2* regulates the expression of *PK* under salt stress conditions, suggesting that the role of PK in the salt stress response may involve an interaction with ENO2, which is the second enzyme in the last step of glycolysis [93]. The demonstrated functions in response to high salinity help confirm the possible roles under the salt stress of genes in this list, which contain a high density of the identified high- and moderate-impact SNPs/InDels.

## 5. Conclusions

The present study examines different rice genotypes that lead to different photosynthetic performances under salt stress conditions during the reproductive stage, which ultimately affects yield components after recovery. Among rice for which the *P*_N_ was correlated with *C*_i_ under salt stress, the HYR had a lower *P*_N_ than the LYR, suggesting that HYR can inhibit salt accumulation to the aerial part via reduced transpiration. Moreover, the whole-genome polymorphisms revealed many genes that contain high- and moderate-impact variants that are over-represented in apoptotic process and kinase activity. Our list of genes that are highly variable and highly expressed in reproductive tissues can serve as a valuable resource for genetic and genomic studies of rice in response to salt stress.

## Figures and Tables

**Table 1:** Correlation between *P*_N_ and other photosynthetic parameters *g_s_*, *C*_i_ and *E*) in the penultimate leaves of each rice varieties at flowering stage grown under normal and salt stress condition for 0, 3, 6, and 9 days.

No.	Variety	Normal						Salt stress					
		*g_s_*		*C*_i_		*E*		*g_s_*		*C*_i_		*E*	
1	Pokkali	0.93	***	0.50	*	0.85	***	0.90	***	0.19	ns	0.87	***
2	Hahng Nahk	0.54	*	0.07	ns	0.36	ns	0.84	***	-0.07	ns	0.73	**
3	Daw Khao	0.82	***	-0.08	ns	0.79	***	0.96	***	0.31	ns	0.96	***
4	Man Wua	0.72	**	0.09	ns	0.74	**	0.87	***	0.18	ns	0.91	***
5	Plah Sew Dam	0.80	***	0.05	ns	0.77	***	0.95	***	0.39	ns	0.98	***
6	E-mum	0.93	***	0.54	*	0.81	***	0.84	***	0.32	ns	0.89	***
7	Rahk Haeng	0.68	**	-0.17	ns	0.82	***	0.70	**	-0.38	ns	0.78	***
8	In Paeng	0.91	***	0.52	*	0.75	***	0.81	***	0.08	ns	0.83	***
9	Sam Ahang	0.39	ns	-0.32	ns	0.07	ns	0.77	***	0.06	ns	0.57	*
10	Ma Yom	0.82	***	0.39	ns	0.82	***	0.90	***	0.72	**	0.94	***
11	Tah Bahn	0.71	**	-0.01	ns	0.63	**	0.86	***	-0.57	*	0.86	***
12	Mahk Yom	0.59	*	-0.33	ns	0.36	ns	0.84	***	0.28	ns	0.81	***
13	Hahng Mah Nai	0.73	**	0.29	ns	0.61	*	0.84	***	0.08	ns	0.83	***
14	Khitom Khao	0.86	***	-0.36	ns	0.74	**	0.89	***	-0.52	*	0.88	***
15	Mahk Bid	0.61	*	0.06	ns	0.10	ns	0.87	***	0.41	ns	0.78	***
16	Leuang Dong	0.97	***	0.51	*	0.88	***	0.86	***	-0.51	*	0.74	**
17	Ruang Diaw	0.91	***	0.37	ns	0.84	***	0.87	***	0.39	ns	0.84	***
18	Mae Mai	0.88	***	0.37	ns	0.83	***	0.96	***	-0.50	*	0.97	***
19	Plah Khaeng	0.85	***	0.32	ns	0.81	***	0.93	***	0.44	ns	0.94	***
20	Jao Khao	0.83	***	-0.28	ns	0.74	**	0.95	***	-0.60	*	0.89	***
21	Muay Hin	0.72	**	0.15	ns	0.76	***	0.87	***	0.20	ns	0.87	***
22	Dawk Mai	0.89	***	0.16	ns	0.84	***	0.94	***	0.40	ns	0.92	***
23	Ta Pow Lom	0.91	***	0.32	ns	0.75	***	0.96	***	-0.46	ns	0.91	***
24	Di Si	0.56	*	-0.38	ns	0.57	*	0.85	***	-0.66	**	0.90	***
25	Med Makham	0.88	***	0.40	ns	0.81	***	0.82	***	-0.63	**	0.84	***
26	Niaw Mali	0.93	***	0.45	ns	0.88	***	0.94	***	0.40	ns	0.95	***
27	Daw Dawk Mai	0.63	**	-0.11	ns	0.65	**	0.86	***	0.44	ns	0.92	***
28	Nahng Nuan	0.87	***	0.49	ns	0.80	***	0.87	***	-0.65	**	0.79	***
29	Sew Mae Jan	0.70	**	0.14	ns	0.78	***	0.92	***	0.56	*	0.93	***
30	Leuang Pratew123	0.89	***	0.41	ns	0.82	***	0.95	***	-0.39	ns	0.95	***

***p-value < 0.001; **p-value < 0.01; *p-value < 0.05; ns, not significant. *P*_N_: Net photosynthetic rate; *g*_s_: Stomatal conductance; *C*_i_: Intercellular CO_2_ concentration; *E*: Transpiration rate.

**Table 2:** Number of sequenced reads and frequency of single-nucleotide polymorphisms (SNPs) and insertions/deletions (InDels) detected in all rice varieties.

Variety	Total Reads	Mapped Locations	Mapping Rate (%)	Number of SNPs		Number of InDels	
				Total	Per 100 kb	Total	Per 100 kb
MY	38,018,919	34,035,049	89.5	707,759	188.8	89,400	23.7
KK	35,704,270	32,986,362	92.4	678,820	181.2	85,700	22.8
JK	31,293,199	29,419,191	94	497,512	132.7	61,385	16.3
NN	33,632,482	31,973,632	95.1	583,761	155.7	73,471	19.5

**Table 3:** List of the top 10 highest-expressing genes in any one of the reproductive tissues.

RAP Id	Description	Chr	Position	Ref	Alt	Sequence Ontology
OS01G0689900	OsWAK10d - OsWAK receptor-like cytoplasmic kinase OsWAK-RLCK	1	28495524	G	T	missense
		1	28495527	C	G	missense
		1	28495528	C	A	missense
		1	28495538	A	C	missense
OS01G0781200	rp1	1	33101161	A	G	missense
OS01G0810600	protein kinase domain containing protein	1	34439934	T	G	missense & splice region
		1	34442278	T	C	missense
		1	34442281	G	T	missense
		1	34442374	A	C	missense
		1	34442388	G	T	missense
		1	34442401	T	C	missense
		1	34442404	T	C	missense
		1	34442433	A	G	missense
		1	34442448	T	A	missense
		1	34442454	C	T	missense
OS01G0836700	GPR107 precursor	1	35869685	T	C	missense
OS02G0127700	phosphoribosyl transferase	2	1439928	AC	A	frameshift
		2	1439888	AGGG	A	disruptive inframe deletion
OS02G0523500	TUDOR protein with multiple SNc domains	2	19100892	A	T	missense
OS03G0124300	receptor-like protein kinase	3	1410653	C	A	missense
OS03G0262300	AT hook motif family protein	3	8596557	A	AGGGGACGGCGAC	disruptive inframe insertion
OS03G0347200	ABH1	3	12984797	G	T	missense
OS03G0800200	PAZ domain containing protein, *OsMEL1*	3	33375066	C	T	splice acceptor
		3	33376480	GAC	G	frameshift
		3	33376483	G	GTA	frameshift
		3	33375166	A	G	missense
		3	33375169	C	A	missense
		3	33375170	G	T	missense
		3	33376283	T	G	missense
		3	33376290	G	A	missense
		3	33376305	C	T	missense
		3	33379671	C	T	missense
OS04G0457800	BRASSINOSTEROID INSENSITIVE 1-associated receptor kinase 1	4	22872259	A	C	missense
		4	22872262	G	C	missense
OS05G0466900	protein kinase family protein	5	22914110	C	T	missense
OS05G0548300	MDR-like ABC transporter	5	27208244	A	T	missense
OS05G0596600	RecF/RecN/SMC N terminal domain containing protein	5	29737585	AAT	A	frameshift
		5	29737590	T	TTA	frameshift
		5	29737576	G	A	missense
		5	29737582	C	T	missense
		5	29737589	C	T	missense
		5	29737620	T	G	missense
		5	29737621	T	A	missense
		5	29737625	T	C	missense
		5	29737629	C	A	missense & splice region
OS06G0116100	CPuORF21 - conserved peptide uORF-containing transcript	6	887012	T	G	missense
OS06G0167500	SHR5-receptor-like kinase	6	3417483	A	G	missense
OS06G0585982	receptor-like protein kinase precursor	6	22953022	A	C	missense
		6	22953076	G	A	missense
OS07G0695400	KIP1	7	29635510	A	G	missense
		7	29636167	A	G	missense
		7	29635792	A	G	missense
OS08G0124100	lectin-like receptor kinase 1	8	1315037	T	C,A	missense
OS08G0190300	NB-ARC domain containing protein	8	5279107	C	G	missense
OS08G0564100	ABC transporter, ATP-binding protein	8	28252908	TC	T	frameshift
		8	28252913	GC	G	frameshift
OS09G0348400	senescence-induced receptor-like serine/threonine-protein kinase	9	10945517	G	A	missense
		9	10945523	A	G	missense
OS10G0151100	OsWAK103 - OsWAK receptor-like protein kinase	10	3065467	G	C	missense
OS10G0346600	vacuolar-sorting receptor precursor	10	10412967	G	C	missense
OS10G0468500	receptor-like protein kinase precursor	10	17314599	G	C	missense
OS11G0148500	pyruvate kinase, *OsPK1*	11	2242566	G	A	stop gained
		11	2242576	AG	A	frameshift
		11	2242326	C	A	missense
		11	2242329	G	A	missense
		11	2242336	G	C	missense
		11	2242338	G	A	missense & splice region
		11	2242516	C	A	missense & splice region
		11	2242584	GAAC	G	conservative inframe deletion
OS11G0227100	NB-ARC domain containing protein	11	6657328	T	A	stop gained
		11	6657183	A	C	missense
		11	6657186	A	G	missense
		11	6657264	T	C	missense
		11	6657340	A	G	missense
		11	6657367	G	A	missense
		11	6657405	A	G	missense
		11	6657435	A	T	missense
		11	6657480	A	G	missense
		11	6657518	C	G	missense
		11	6657528	G	A	missense
		11	6657533	G	T	missense
		11	6657537	A	G	missense
OS12G0102500	senescence-induced receptor-like serine/threonine-protein kinase	12	119480	G	C	missense
		12	119522	C	T	missense
OS12G0197500	SGS3	12	5038711	T	C	missense
OS12G0197700	leafbladeless1	12	5049404	C	T	missense
		12	5049448	C	T	missense
		12	5049449	A	G	missense

## Supplementary Materials

The followings are available online at https://www.mdpi.com/2073-4425/10/8/562/s1, Figure S1: Venn diagram showing the number of SNPs (A) and InDels (B) between LYR (MY and KK) and HYR (JK and NN)., Figure S2: Expression level of 378 enriched genes in the post-emergence inflorescence, pre-emergence inflorescence, anther, pistil and panicle of rice, Figure S3: Gene structures and locations of high- and moderate-impact SNPs/InDels identified in MY/KK-JK/NN of A) *OsXA21* B) *OsMEL1* and C) *OsPK1*. Brown and green boxes indicate 3′- or 5′-UTRs and CDSs, respectively., Figure S4: Photosynthetic parameters under normal (A, C, E, G) and salt stress (B, D, F, H) conditions of each rice variety at the flowering stage: net photosynthesis rate, *P*_N_ (A, B); stomatal conductance, *g*_s_ (C, D); intercellular CO_2_ concentration, *C*_i_ (E, F); and transpiration rate, *E* (G, H). Bar represents the standard error of four replicates. ANOVA was performed, followed by mean comparison with DMRT. Different letters above the bars show a statistically significant difference in means at *p*-value < 0.05. MY: Ma Yom, KK: Khitom Khao, JK: Jao Khao, NN: Nahng Nuan., Figure S5: Number of filled grains per plant under normal and salt stress conditions of each rice variety at the flowering stage. Bar represents the standard error of four replicates. ANOVA was performed, followed by mean comparison with DMRT. Different letters above the bars show a statistically significant difference in means at *p*-value < 0.05. MY: Ma Yom, KK: Khitom Khao, JK: Jao Khao, NN: Nahng Nuan. Table S1: List of rice varieties used in this study, Table S2: List of rice varieties and phenotypic data under normal and salt stress conditions of each rice variety at the flowering stage., Table S3: Number of SNPs and InDels identified in HYR and LYR varieties. SNPs and InDels were annotated based on their location, Table S4: Annotation of SNPs and InDels defined in specific, shared and common variants., Table S5: Frequency of SNPs and InDels identified in LYR and HYR that were detected on individual rice chromosomes., Table S6: List of 2089 genes identified in MY-KK/JK-NN containing high- and moderate-impact variants, Table S7: Summary of gene ontology enrichment analysis, Table S8: Variation in 262 genes identified in MY/KK-JK/NN to be expressed in reproductive tissues.
