# Supplementary material for: Comparative Genomic Analysis of Rice with Contrasting Photosynthesis and Grain Production under Salt Stress
Source: Genes (Basel). 2019 Jul 25;10(8):562. doi: 10.3390/genes10080562 (PMC6722916; doi:10.3390/genes10080562)
Supplement: Supplementary file 1 [file genes-10-00562-s001.zip › All Supplementary Files/Fig.S3.pdf]

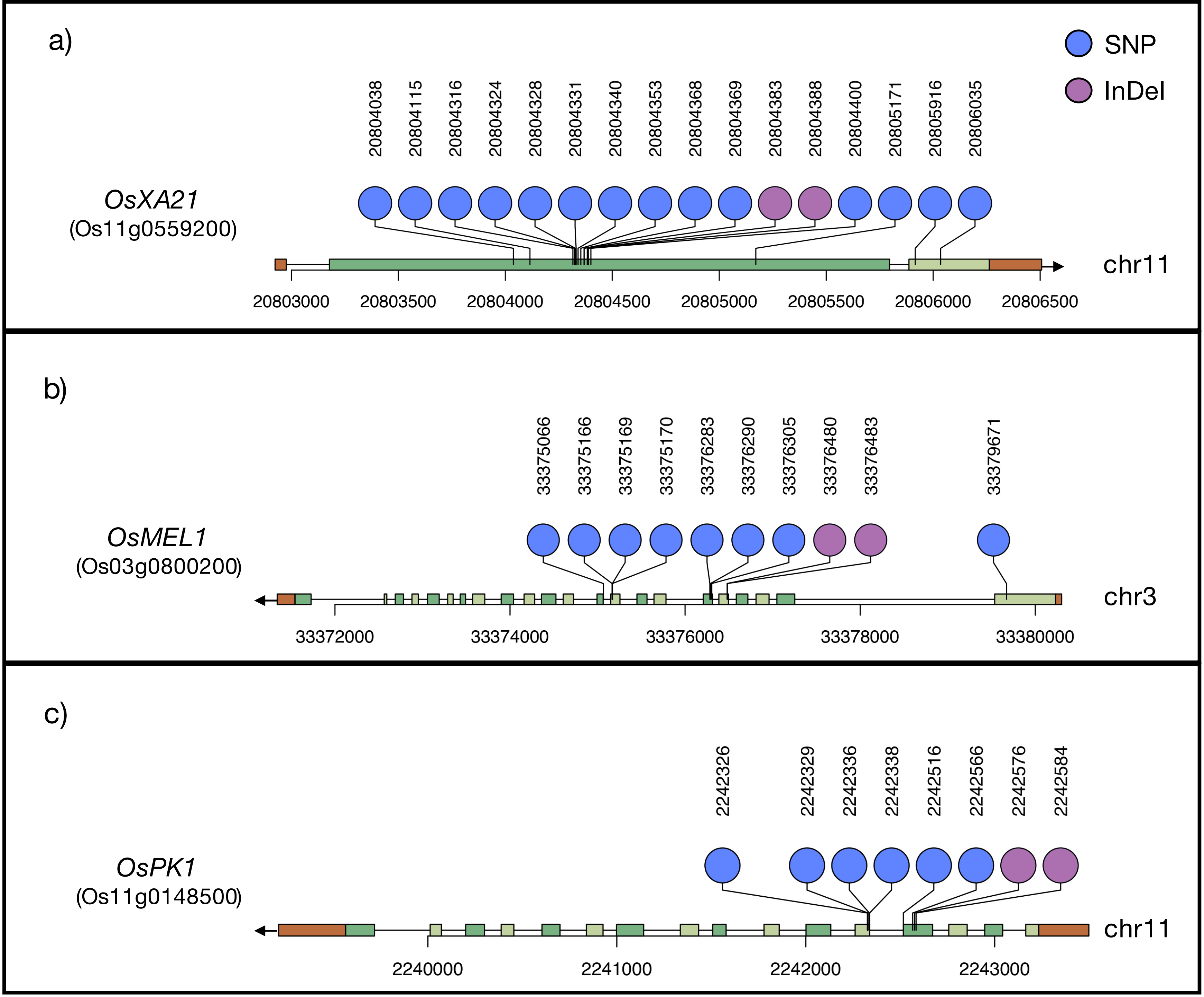

Fig. S3 Gene structures and locations of high- and moderate-impact SNPs/InDels identified in MY/KK-JK/NN of A) *OsXA21* B) *OsMEL1* and C) *OsPK1*. Brown and green boxes indicate 3'- or 5'-UTRs and CDSs, respectively.
