# Supplementary material for: Comparative Genomic Analysis of Rice with Contrasting Photosynthesis and Grain Production under Salt Stress
Source: Genes (Basel). 2019 Jul 25;10(8):562. doi: 10.3390/genes10080562 (PMC6722916; doi:10.3390/genes10080562)
Supplement: Supplementary file 1 [file genes-10-00562-s001.zip › All Supplementary Files/Fig.S4.pdf]

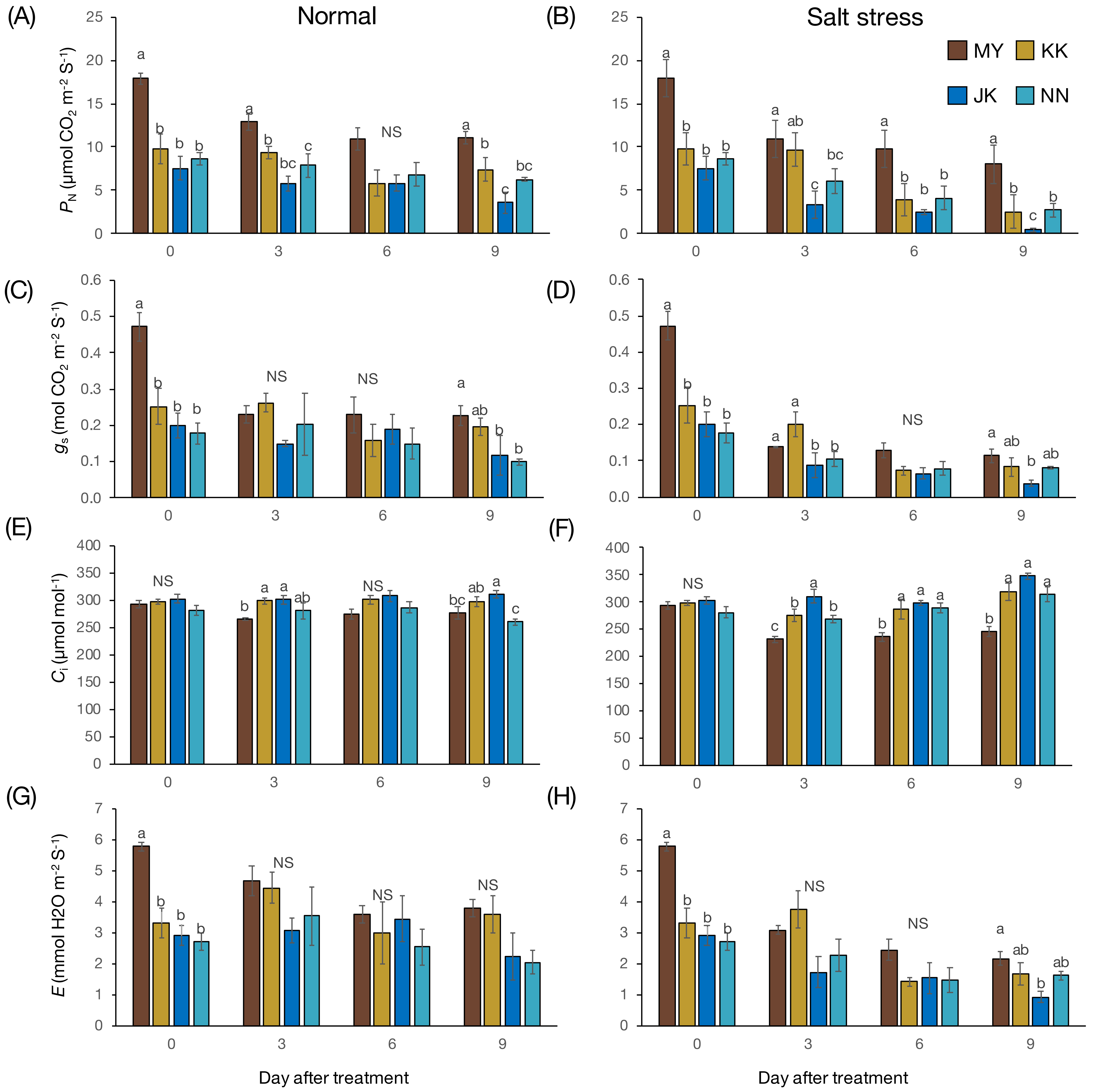

Fig. S4 Photosynthetic parameters under normal (A, C, E, G) and salt stress (B, D, F, H) conditions of each rice variety at the flowering stage: net photosynthesis rate, P_N (A, B); stomatal conductance, g_s (C, D); intercellular CO_2 concentration, C_i (E, F); and transpiration rate, E (G, H). Bar represents the standard error of four replicates. ANOVA was performed, followed by mean comparison with DMRT. Different letters above the bars show a statistically significant difference in means at $p<0.05$. MY: Ma Yom, KK: Khitom Khao, JK: Jao Khao, NN: Nahng Nuan.
