# Supplementary material for: Comparative Genomic Analysis of Rice with Contrasting Photosynthesis and Grain Production under Salt Stress
Source: Genes (Basel). 2019 Jul 25;10(8):562. doi: 10.3390/genes10080562 (PMC6722916; doi:10.3390/genes10080562)
Supplement: Supplementary file 1 [file genes-10-00562-s001.zip › All Supplementary Files/Fig.S5.pdf]

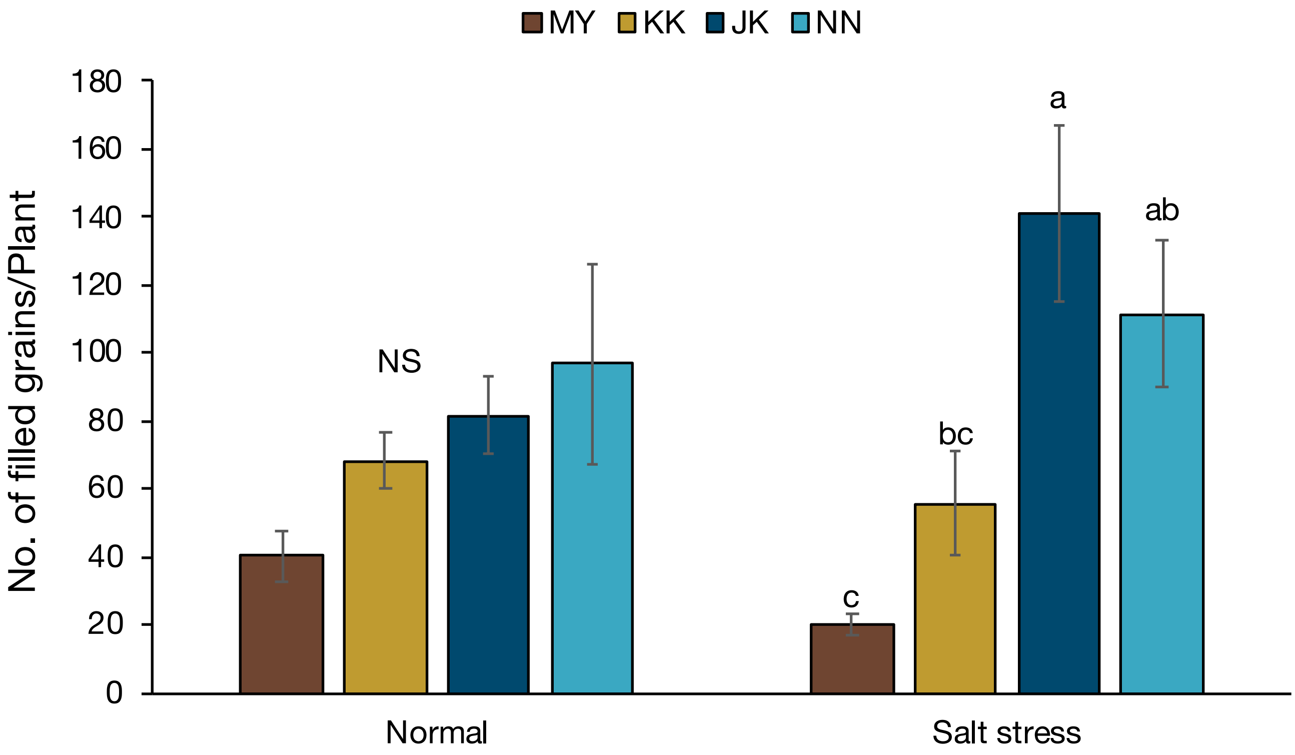

Fig. S5 Number of filled grains per plant under normal and salt stress conditions of each rice variety at the flowering stage. Bar represents the standard error of four replicates. ANOVA was performed, followed by mean comparison with DMRT. Different letters above the bars show a statistically significant difference in means at $p<0.05$. MY: Ma Yom, KK: Khitom Khao, JK: Jao Khao, NN: Nahng Nuan.
