# Supplementary figures and images for: Comparative Genomic Analysis of Rice with Contrasting Photosynthesis and Grain Production under Salt Stress
Source: Genes (Basel). 2019 Jul 25;10(8):562. doi: 10.3390/genes10080562 (PMC6722916; doi:10.3390/genes10080562)

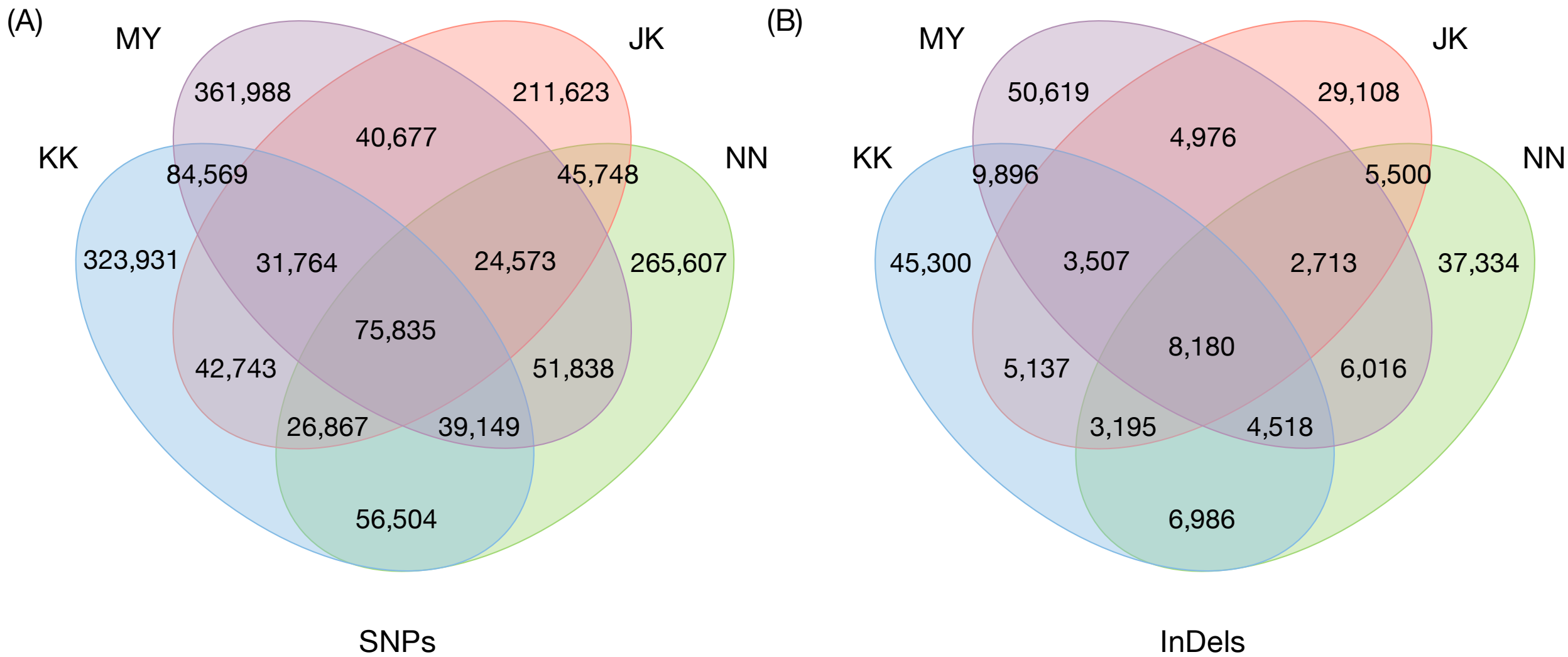

**Fig. S1** Venn diagram showing the number of SNPs (A) and InDels (B) between LYR (MY and KK) and HYR (JK and NN).

Supplement: Supplementary file 1 [file genes-10-00562-s001.zip › All Supplementary Files/Fig.S1.pdf]

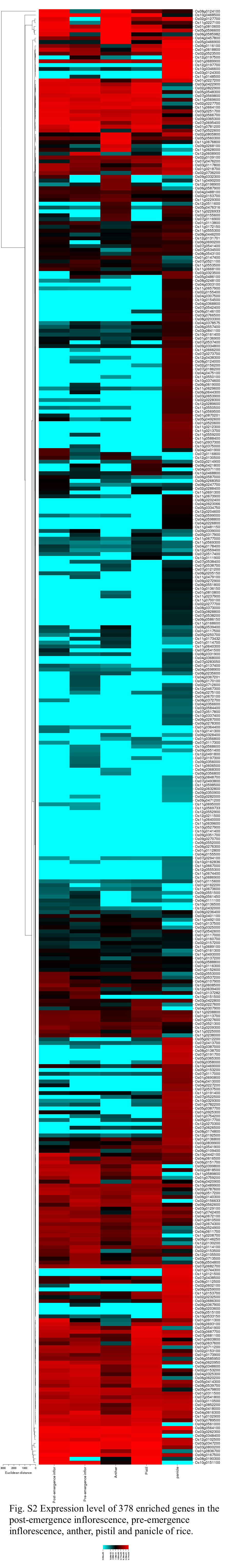

Supplement: Supplementary file 1 [file genes-10-00562-s001.zip › All Supplementary Files/Fig.S2.jpg]
